# A Sketch of the History of the Use of Blood-Letting as a Remedy for Disease
*This is one of the few medical subjects which have not been treated of in this Journal in a historical, as well as in a theoretical and practical, manner; this sketch will, then, we consider, be received with pleasure by our readers, as an essay to supply the deficiency on the subject of blood-letting.—Edit.


**Published:** 1819-11

**Authors:** 


THE LONDON
### Medical and Physical Journal.

5 OF VOL. XLII.] NOVEMBER, 1819. [NO. 249.

"For many fortunate discoveries in medicine, and for the detection of numerous "errors, the world is indebted to the rapid circulation of Monthly Journals; "and there never existed any work to which the Faculty in EUROPE and "AMERICA were under deeper obligations than to the Medical and Physical "Journal of London, now forming a long, but an invaluable, series." RUSH.

### Original Communications.

FOR THE LONDON MEDICAL AND PHYSICAL JOURNAL.

### *A Sketch of the History of the Use of Blood-letting as a Remedy for Disease.** By Δ.

IN tracing the history of the use of blood-letting as a remedy for disease, we can rise only to the source whence the nations of Europe, thirty ages since, derived the origin of their knowledge in science as well as in the arts, and which, successively cultivated by ensuing generations, has raised us to a degree of eminence in the scale of intellectual nature that has probably never been excelled. It is from the Egyptians, through the medium of the Greeks, that we derive the use of this, the most powerful of all our measures for the alleviation of disease.

The invention of the processes of most of the arts has, in the eyes of barbarous nations, appeared so wonderful an effort, that they have in general either attributed them to supernatural powers, or to imitation of the instinctive actions of brute animals, which have been considered to be under the immediate direction of similar agents. Thus, the origin of the use of blood-letting is referred by Egyptian tradition to the *hippopotamus*, who, when sick, is said to seek a sharp-pointed reed, with which he wounds a vein of one of his limbs, and thus induces an emission of blood. But it is more probable that the practice originated from the same source as nearly the whole of the other instances of the relative power of man over the operations of nature,—inductive and analogical reasoning. Men, observing the good effects of spontaneous evacuation of blood in various diseases, were thence induced to imitate them by art.

* This is one of the few medical subjects which have not been treated of in this Journal in a historical, as well as in a theoretical and practical, manner; this sketch will, then, we consider, be received with pleasure by our readers, as an essay to supply the deficiency on the subject of blood-letting.—EDIT.

The earliest account of the use of this remedy is conveyed to us by the writings of STEPHEN of Byzantium, who relates that PODALIRIUS, on returning from the siege of Troy, was thrown on the coast of Caria, where he relieved SYRNA, the daughter of king DAMÆTHUS, from the ill consequences of a fall from a house, by bleeding her in both arms: and, what seems to show the novelty of the practice at that period, the physician received the hand of Syrna as the reward of his services. A blank of seven hundred years then ensues in the history of the application of this measure; but, after that interval, we find the records of it in the works of HIPPOCRATES.*

Hippocrates speaks of blood-letting as a common remedy, and of its being effected by means of scarification and the cupping-glass, as well as by opening arteries and veins. The Grecian physicians probably derived the use of the former measure also from the Egyptians, whom HERODOTUS reports to have adopted it, as well as PROSPER ALPINUS, who also gives a description of the instruments they employed in its execution. But we also trace the use of this mode of blood-letting in other civilized nations of antiquity, and amongst many uncultivated savage tribes. In the way of *acupuncturation*, it was resorted to at a very remote period by the Japanese and Chinese; and is commonly employed by the natives of America† and by the Hottentots.‡

Believing certain vessels to be particularly connected with certain viscera, much importance was attributed by Hippocrates to the choice of the vein, as well as the region of the body from which the blood should be drawn. In general, a convenient one as near as possible to the seat of the disease was chosen; in the

---

* HERODICUS, who preceded Hippocrates, seems to have confined the art of medicine to regulations of gymnastic exercise. He observed the extraordinary strength of body acquired by those who displayed their powers in the olympic games; and that those who had been sickly and feeble, often by those measures acquired health and vigour. PLATO also relates, that Herodicus had himself suffered a disease considered to be incurable, from which he had been delivered by practising the exercises of his own academy, (for he had previously been the director of a palestrum:) from this time he devoted his whole attention to the regulation of a system of gymnastic exercises, solely of medical application. GALEN says, that his rules were in many respects well adapted for prophylactic means, and bore a proper relation to the temperament, age, sex, climate, and season, attendant on the disease to which they were to be applied. He seems, however, to have been led into some excesses by his enthusiasm for his favourite measure; for a common direction to his patients was, to go from Athens to Eleusis, passing by Megara, a distance of nearly fifty miles, without either rest or food.

† See *Dampier's Voyages.*

‡ See *Histoire générale des Voyages*, tom. xviii. c. 14. See also *Mantissa schematica de Acupunctura* (cum icone instrumenti); *ad Dissert. de Arthritide*, auctore G. RHYNE, 8vo. Londini, 1683; and *De Puncto, Dissert.* Lugdun. Batav. auct. J. BIDLOO, 1709. For the observations of HIPPOCRATES on scarification and cupping, see his book Περὶ Ἰητρεῦ, § vi. and vii.

superior extremities, when the malady was above the division of the body formed by the diaphragm; and in the lower extremities, in those below it. In inflammatory affections of the throat, he opened the veins of the tongue; and in pains of the head, vertigo, &c. thôse of the forehead, or on the sides of the nose. The basilic vein correspondent with the affected side, was chosen in pleurisy; and the same vessel of the left arm, in diseases of the spleen. The blood, in inflammatory affections, was generally permitted to flow until it changed in colour,—that is, assumed the florid hue;* or until syncope was produced. It was resorted to in almost all violent pains, inflammations, and ardent fever, when the age and strength of the patient permitted.

The most distinguished of the immediate successors and disciples of Hippocrates, Diocles, chief of the dogmatic sect, followed in general the precepts of his master in the use of this remedy; but subsequent physicians did not so readily forego the doctrines of their hypotheses, to be guided in practice by the results of experience. Chrysippus of Gnidos abrogated the use of blood-letting; and the most celebrated of his sectaries, Erasistratus, rarely resorted to it except in cases of hæmorrhage: yet Erasistratus was rather inclined to follow the conduct of the Empirics, had made several discoveries in anatomy, and first formed rational notions respecting the nature of inflammation, which he attributed to increased action of the small arteries; and this he also considered to be the essential origin of fever. To avoid the use of blood-letting, he had recourse to ligatures to the extremities, very restricted diet, emetics, and purgatives; and gymnastic exercises.

But a revolution again ensued, from the authority of a man whose glory is coæval with the subsequent records of the history of his art. Herophilus restored the use of bleeding in inflammations, especially those of the throat and thoracic organs, and in many diseases accompanied with much severity of pain; but his practice in this respect seems to have been timid, though guided by the precepts of some celebrated empirics, particularly Acron, Serapion, and Philinus. This was the time of the chief reign of what may be termed rational empiricism. Those physicians, and their disciples, took much pains to graduate the doses of remedies; they witnessed their administration, and chose the most appropriate periods for doing it; and carefully directed the regimen that should be followed under their operation. They considered that it was sufficient that experience had pointed out the appropriate medicines, to lead to the

* This almost universally takes place, after from ten to fifteen or twenty ounces have been drawn from a vein in a moderate-sized stream.

administration of them to their patients: they left reasoning to the dogmatists; saying, that chance led to the discovery of remedies; that different trials of them had established their use, which had been preserved by tradition; and that comparison, and the analogy and relations discerned between a known and an unknown disease, might serve for guides to physicians in extraordinary cases. Such also was the method of HERACLIDES of Tarentum; who, according to Galen, never made an assertion contrary to truth, even to defend the interests of his sect, and who never related what he had not himself observed. Heraclides, however, employed bleeding more freely than his immediate predecessors: he commonly resorted to it in phrenitis, cynanche, epilepsy, gout, syncope, and the diseases of the heart in general.

The course of this history now leads us to a very important epoch in the annals of our art,—that of the first peaceable establishment of a physician at Rome in the person of ASCLEPIADES, a reformer of medical theory hardly inferior in celebrity to HIPPOCRATES, GALEN, or even BOERHAAVE.

Asclepiades was a native of Prusia in Bythinia. He first appeared in Rome as a teacher of rhetoric, but he soon devoted himself solely to the practice of medicine; and here his powerful genius triumphed over every obstacle. He formed a new system of doctrine, composed of a selection from the principal dogmas of the different sects which had flourished in Greece, embellished by all the splendour with which genius and eloquence could adorn them; and the people that an age previously had expelled Archagatus from their city, now became justly proud of a man who made Rome the centre of knowledge of this branch of science, and overshaded even the glory of the founder of the Cosian school. But Asclepiades owed much of his reputation to the fortunate situation in which he was placed, and to his powers as a logician; though some of his physiological views were accurate and original, and several of his dogmas have been advanced under different forms at various succeeding periods, and are admitted at the present day. He believed the chief causes of disease to consist in obstruction of the minute vessels of the body; and that, when this congestion amounted to a considerable extent, inflammation was the result: active exercise was here his chief remedy. He also maintained, that one diseased action is to be averted by the interposition of another. These notions, carried to excess in their application, led him to resort but little to the use of blood-letting. He recommended exercise on horseback even in ardent fevers, and an abstinence from all liquids during the first few days of the disease, that the strength of the body might be exhausted, and depletion of the vessels be thus effected. He only employed bleeding in cases of intense pain, convulsions, and hæmorrhage. The practice of Asclepiades was congenial to the character of the people amongst whom he resided, and he indeed appears to have modelled his conduct in every respect to their own taste. For those physicians who were not very anxious to acquire a knowledge of the primitive causes of life, health, and disease; who took nature for their guide; contenting themselves with attentively observing the progress of disease, without attempting to interfere with its course when it ran through its different stages with precision, only endeavouring to restore it to its natural regularity when it deviated from that order, Asclepiades had the most profound contempt, and he facetiously termed their conduct,—*a meditation upon death.*

The reign of the opinions and method of practice of Asclepiades was not of long duration, although his mechanical notions respecting physiology had drawn over to his party the sectaries of Epicurus and Leucippus, at that time the directors of public opinion in physics in Greece and Rome. A sect sprang up, the founder of which was Themison, whose doctrines gave more scope to the play of an active imagination, and were more in harmony with the rising taste of the people. It seems ever to have been the fate of medicine to have the basis of its doctrines deduced from the favourite branch of physical science of the concurrent period. Music seems to have furnished Themison with the first ideas of his system: *constricted fibres, relaxed fibres,* and (what it is difficult to form any precise idea of) a *combination of those two states,* constituted the nature of the various species of disease. This doctrine has also been since renewed at various times under various forms, with all the pomp of a new theory; and is indeed applied at the present day to the doctrine of fever, by a physician of considerable reputation. This sect termed themselves the methodists. Their practice was in general simple: consisting in the use of *blood-letting,* and some other analogous means, to relax the inordinate tone of constricted fibres; and of stimulants, to increase that of relaxed fibres; but in diseases of the third class, and in those of long duration, they were confused and indecisive in their conduct, and they endeavoured to hide their embarrassment by cabalistic arts. Bleeding was here but rarely employed: their grand measure was what they termed the *metasyncritick* or *resumptive circle.* The introduction of the use of *leeches* seems to have been due to Themison; they were first spoken of as being employed for blood-letting in his works; and, as he was not much inclined to the abstraction of blood, he probably thought by this measure to alleviate local constriction, without any considerable loss of that fluid.

The doctrines of this sect were prevalent until the time of

Celsus. Blood-letting was then again resorted to with more freedom, though with much caution in its use; and, perhaps, still with too much reserve in its application, since old age, pregnancy, and infancy, were designated as contra-indications to its employment. There were however but few diseases in which Celsus did not employ it, occasionally, in their first stages; though in acute maladies it was not resorted to, in general, after the third or fourth days, and was but rarely carried to such an extent as to induce syncope,—a consequence which he seemed to regard rather with an undue degree of fear; though, perhaps, he might have been led to this by the rash conduct of some of the remaining dogmatists.

In continued and epidemic fevers, he placed his chief reliance in this remedy, referring the use of purgatives and abstinence, the common measures of his immediate predecessors in all fevers, to agues. Severe pain, hæmorrhage, convulsions, and palsy, always claimed the application of this measure, in his opinion. He never used it in the paroxysms of fever, and always stopped the flow of blood when it assumed the florid hue.

Coelius Aurelianus succeeded him: he principally adopted the doctrines of Themison and his sectaries, which he has developed in a comprehensive manner, though his style is obscure and inelegant. He employed blood-letting as a common measure according to his theoretical principles, and consequently in too partial and restricted a manner; though he generally employed it in the early stage of fevers.

Aretæus, who was contemporary with, or perhaps somewhat antecedent to, Galen, and whom we first notice, that this part of our history may terminate with the view of the doctrines which reigned universally throughout Europe for so many ages, employed blood-letting much more freely, especially in ardent fever; though he also adopted the notions of *strictum* and *laxum*. He recommended that it should be taken from a large orifice. He conformed to the notions of Hippocrates respecting the importance of the choice of vessels, which appear not to have been much regarded by his immediate predecessors. Thus, he abstracted blood from the region of the pubes in affections of the uterus, from the forehead in those of the brain, and from the tongue in cynanche. He also appears to have used arteriotomy more frequently than ordinary; and very commonly employed cupping with scarification.

We now arrive at Galen. He was a native of Pergamus, studied at Alexandria; and, at the age of 32, went, in the 193d year of the common æra, to Rome. Let us here adduce the character given of this celebrated man by one of the finest geniuses that appeared to enlighten human nature in the latter part of the last age: this will form an appropriate proëmium to the account of the revolution that took place in the use of the remedy forming the express subject of this history, from the system of doctrine he instituted.

"After the lapse of many ages lost to its progress, after many disputes and errors, medicine had need to seek a new and more certain road. It was time for it to return from the following of dogmas, to the contemplation of nature, or of Hippocrates, her faithful interpreter. Galen appeared. His genius was sufficiently vast to embrace all the sciences, and to cultivate them all with equal success. From his infancy he gave proofs of his rare capacity, and even in the schools he began to discern the vacuity of the reigning systems. But little satisfied with what his masters taught him as incontestible truths, as the eternal principles of the art, he perused Hippocrates: he was enlightened, as it were, with a new beam. On comparing his descriptions with nature, his astonishment and admiration were increased. Hippocrates and nature were from that time the only masters from whom he would receive instructions. He began to comment on the writings of the father of medicine: he presented his views under different aspects, which had not been previously discerned; he repeated his observations, enriched them, and supported them with all that science and philosophy could lend, either by analogous facts, a comparison of different theories, or the combination of different modes of reasoning. In a word, Galen resuscitated hippocratic medicine, and gave to it a splendour it did not possess in its primitive and simple state. But, it must be acknowledged, what it acquired in his hands, was perhaps rather showy adornments than true riches. The observations collected, and the rules traced, by Hippocrates, on assuming a more brilliant and systematic character, lost much of their purity: under the extraneous envelopments derived from the sciences, or from different dogmas, nature, whom the physician of Cos had always embraced with so much propriety and reserve, was almost stifled and lost; and the art, surcharged with rules, either superfluous or too subtile, only became embarrassed with many difficulties not really attendant on nature." This is the description of CABANIS.

The knowledge of anatomy which Galen had acquired at Alexandria, application to which he never ceased to pursue in the dissection of brute animals and in the works of ARISTOTLE, contributed to render his knowledge of the functions of the animal economy more precise than that of his predecessors. He also caught a glimpse of the opinions of Aristotle respecting the two series of functions of animal bodies; notions that afterwards remained neglected until they were discerned by BUFFON, and finally fully developed by BICHAT, under the designation of the systems of organic and animal life. All this gave a boldness and decision to his measures; and his theoretical opinions leading him to the common use of blood-letting, this remedy was consequently, under his direction, carried to a great extent. He is the first who commonly speaks of the quantity he abstracted; and this was on many occasions very great; often carrying it so far as to induce syncope, which he nevertheless considered a hazardous measure. There were few diseases in which he did not sometimes employ it, in persons above the age of puberty, and when the strength would permit. He was in this respect much regulated by the state of the pulse, though his conceptions on this subject appear to modern physicians to have been merely flights of imagination not applicable to nature. He was careful in the choice of certain veins for certain diseases, generally following the precepts of Hippocrates; and there were few that were not subjected to his lancet and cautery. His epistle to the sectaries of Asclepiades, on the use of blood-letting in fever, and his observations on this subject in his book on Method, contain the substance of his doctrines on this point, and comprise many judicious and highly valuable remarks, and some interesting cases. We there find that he employed blood-letting, even to fainting, in a case of low typhous fever, and with the most fortunate results. This part of his works would be alone sufficient to induce us to join in the eulogia that have been conferred on that great man; though it must not be concealed, much of his glory in the early ages of the modern nations of Europe, arose from some fortunate and adventitious circumstances. His genius was of the species best calculated to ensure his fame: it was active, bold, and penetrative; with a fire that, when employed in censure, made his adversaries shrink from before him. He adopted the philosophy of Aristotle, with some of the metaphysical principles of Plato; and from this he became patronized by the primitive fathers of the Christian church, who also followed it; and, finally, his method, manner, and acquirements, all concurred with those adapted to the taste of the gothic schools.

The use of blood-letting is again becoming carried to a great extent, in diseases to which it was a few years since considered inapplicable. Let the most ardent votaries of this remedy, then, direct their attention to Galen; they will in him find one who will go hand in hand with them to their utmost extremities, and be no more deterred than themselves from having recourse to it, in spite of debility and all its attendant signs, when this arises from local inflammation or partial concentration of the vital powers.

[*To be continued.*]

